# Drivers’ Visual Perception Quantification Using 3D Mobile Sensor Data for Road Safety

**DOI:** 10.3390/s20102763

**Published:** 2020-05-12

**Authors:** Kanghee Choi, Giyoung Byun, Ayoung Kim, Youngchul Kim

**Affiliations:** 1 KAIST Urban Design Lab, Department of Civil and Environmental Engineering, KAIST, Daejeon 34141, Korea; fascino4008@kaist.ac.kr (K.C.); rain8496@kaist.ac.kr (G.B.); 2 KAIST IRAP Lab, Department of Civil and Environmental Engineering, KAIST, Daejeon 34141, Korea; ayoungk@kaist.ac.kr

**Keywords:** visibility, visual perception, point cloud, driver’s safety

## Abstract

To prevent driver accidents in cities, local governments have established policies to limit city speeds and create child protection zones near schools. However, if the same policy is applied throughout a city, it can be difficult to obtain smooth traffic flows. A driver generally obtains visual information while driving, and this information is directly related to traffic safety. In this study, we propose a novel geometric visual model to measure drivers’ visual perception and analyze the corresponding information using the line-of-sight method. Three-dimensional point cloud data are used to analyze on-site three-dimensional elements in a city, such as roadside trees and overpasses, which are normally neglected in urban spatial analyses. To investigate drivers’ visual perceptions of roads, we have developed an analytic model of three types of visual perception. By using this proposed method, this study creates a risk-level map according to the driver’s visual perception degree in Pangyo, South Korea. With the point cloud data from Pangyo, it is possible to analyze actual urban forms such as roadside trees, building shapes, and overpasses that are normally excluded from spatial analyses that use a reconstructed virtual space.

## 1. Introduction

Recently, the number of traffic deaths in Korea has steadily decreased from 5870 people in 2008 to 4185 people in 2017, but the number of accidents has remained almost unchanged, with 218,822 in 2008 and 216,353 in 2017 [1]. Previous traffic safety policies have contributed to reducing the risk of accidents by limiting the speed of traffic and designating child protection zones, but such measures have not been effective in reducing accidents. In this study, we propose a method to reduce traffic accidents on roads through efficient regulation by identifying accident-prone locations instead of using an overarching regulation at the city scale. In particular, we sought to develop an assessment method to evaluate actual urban environments, including various objects on and near roads that affect the visual perception of drivers. We defined a new Euclidean geometric visual perception model here, considering driver cognitive behavior characteristics. In addition, visibility analysis was conducted here using the line of sight (LoS) method, which is based on raycasting and is used in the fields of urban science and geography. Then, we applied the proposed method to three-dimensional (3D) mobile mapping data to find accident-prone locations with large differences in cognitive quantity in a city.

## 2. Background

Accident-prone locations are geographical locations where traffic accidents are concentrated [2]. Many scholars have sought to determine accident proneness in advance by studying the relationship between road geometry and traffic safety. Ahmed et al. [3] found that the geometry of a road is strongly related to traffic accidents, and that the steeper roads are, the higher the traffic accident rate. The average speed of a vehicle, headway time, and headway distance are also affected by the road geometry [4]. Karlaftis and Golias [5] quantitatively measured the impact of road geometry by analyzing road geometry and accident rates through a hierarchical tree structure regression model and predicted accident rates on local roads. In addition, Moradkhani et al. [6] proposed a method to predict accident-prone locations through techniques that match the locations of accidents and the geometry of roads. These studies only focused on linking the actual road geometry with the number of traffic accidents without considering the information that the driver visually obtains.

During driving, the driver generally obtains information through vision [7,8]. Therefore, the visual information obtained by the driver is directly related to traffic safety [9]. To determine the visual information obtained during driving, researchers in the field of computer science have tried to quantitatively measure the visual perception of drivers. Altunm [10] calculated the visual perception of drivers by analyzing images obtained through a camera attached to the vehicle using the image fusion method. Yu et al. [11] developed a driver visual lane model to calculate the road alignments perceived by the driver. However, these scene-based visibility analysis methods (such as image-based or photograph-based methods) are not sufficient for analyzing a driver’s cognitive behavior, which changes according to the situation.

It is important to understand the behavior of the driver in order to establish a desirable and appropriate traffic safety policy. In the perceptual psychology area, various studies have been conducted to analyze drivers’ visual behaviors, and empirical studies have mainly been conducted through driver experiments [12,13,14,15]. In this case, as more data are obtained, the analysis becomes increasingly reliable, but it is difficult to set various conditions and secure many samples. It is also difficult to remove external variables other than the conditions that are assumed in the experiment.

The development of computing technology has focused on quantifying and measuring universal human visual perception. There are two methods of analyzing information obtained via visual perception, namely, directly analyzing an image or applying a geometric visual model. The method of analyzing visual perception information through images involves quantifying human visual perceptions by analyzing the color and depth values obtained from an image. However, this method often displays poor objectivity in continuous environments or three-dimensional environments. In addition, there is a limit to real-time interpretation with respect to cognition in this instance.

Geometric visual models can simulate human visual behaviors. In this way, it is easy to interpret the driver’s visual form in real time. Gibson [16,17] noted that the visual space must be geometrically defined to match our perception to the physical world. Thomas Reid [18] defined such a visual space as forming a spherical geometry, as recently supported by various studies [19]. In human vision, the amount of information that can be obtained with a short glance without head movement is limited. This visual area is called the useful field of view (UFOV) [20]. The driver’s UFOV generally decreases with age [21] and can be characterized by a narrow viewing angle and a long sight distance as the driver’s speed increases [22,23]. Therefore, it is necessary to consider the driver’s UFOV when designing roads in a city for safety.

In order to geometrically define a driver’s vision, initial studies have mainly focused on only the geometry of the road [24,25,26,27]. In this case, it is difficult to consider obstacles such as buildings that affect the driver’s vision in a city. To overcome these limitations, researchers have used geographic-information-system (GIS) data to analyze the visible space by considering more diverse obstacles [28]. However, it is difficult to construct the various urban elements that drivers experience in virtual reality based on GIS data alone. That is, surrounding elements that act as obstacles in the visual field in actual cities are often ignored, e.g., buildings, trees, and signs. In this study, we use 3D mobile scanned data for driver-oriented visual analysis in a near-realistic driving environment. While previous studies investigating driver’s field of view (e.g., [24,25,26,27,29,30]) have mainly focused on only the geometry of road, this study sought to include a visual assessment of urban objects and environments on and near roads. Various urban objects (including trees, surrounding buildings, and entrances to apartment complexes) affect visual perception on roads and provide drivers with various confusing sources of information that are difficult to construct in virtual and conventionally digitized environments.

## 3. Model Development

The basic assumptions of our visual perception model are as follows. First, the basic form follows a hemispherical geometry with a limited vision range. The UFOV, which is a visual area recognized momentarily by the driver, is determined by the horizontal visual angle and the vertical visual angle. The driver’s UFOV varies with speed (as the speed increases, the visual angle decreases). For simplicity, it is assumed that all humans have the same UFOV range and recognition ability. The faster the speed of travel is, the smaller the field of view and longer the LoS. In addition, the vertical visual angle $\theta_{elevation}$ is set to a constant value to focus only on the horizontal visual angle $\theta_{azimuth}$ without considering the size of the vehicle. Therefore, the assumptions applied for the visual perception model in this study are summarized as follows:

1. A typical visually perceived space has a hemispherical geometry.
2. The parameters of the driver’s visible space are the visual angles and sight distance, but the vertical visual angle is fixed.
3. The faster the speed of travel, the smaller the visual angle and the longer the sight distance.
4. Regardless of the vehicle speed, the maximum value of the information that is instantaneously obtained is constant. Therefore, we focused on the ratio of perception.

### 3.1. Definition of Visibility Analysis

**Figure 1:**
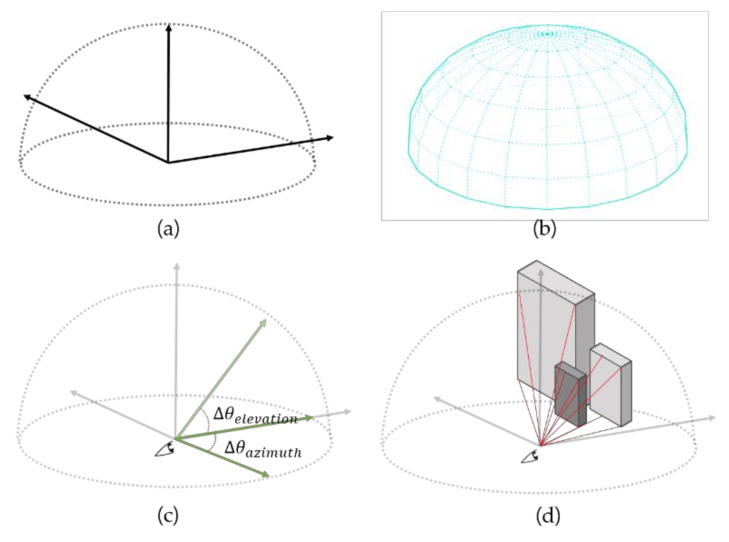
Visible space for a stationary state. (**a**) Human field of view at standstill; (**b**) splitting the visual field based on the unit line of sight; (**c**) line of sight variables; (**d**) recognition of an object with the line of sight method in the visual field.

We sought to measure drivers’ perceived visibility through the degree of detection associated with a driver’s UFOV. The implemented method casts a set of LoSs from the driver’s position. Thus, the defined visible space should be replaced with an LoS. First, as described above, the visible space of the stationary state is a hemispherical geometry, as shown in Figure 1a. We divided the space into certain sections and generated a grid, as shown in Figure 1b. As shown in Figure 1c, each LoS is defined by its vertical and horizontal angles and sight distance. The raycasting of the lines identifies the object in the defined area in Figure 1d.

**Figure 2:**
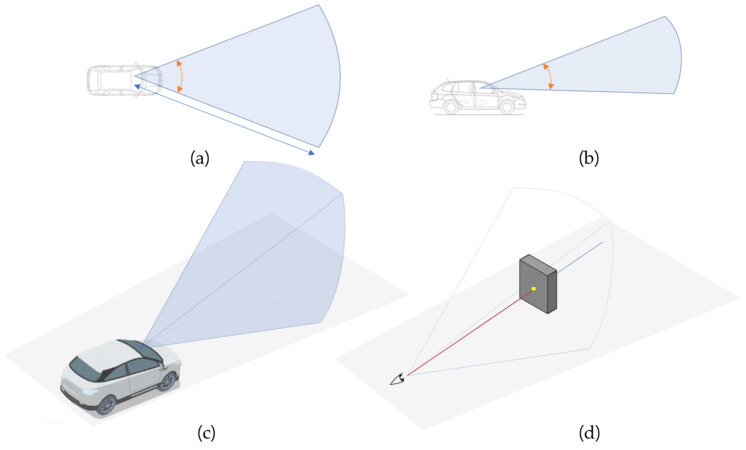
Driver’s visually perceived space. (**a**) Horizontal view of the driver useful field of view (UFOV); (**b**) vertical view of the driver UFOV; (**c**) stereoscopic view of the driver UFOV; (**d**) the line of sight within the driver UFOV.

One visually perceived space can be defined by a horizontal visual angle $\theta_{elevation}$, a vertical visual angle $\theta_{azimuth}$, and a sight distance, as shown in Figure 2a,b. An illustration of a driver’s visually perceived space in three dimensions according to the determined variables is shown in Figure 2c. Within this field of view, the LoS is adjusted by recognizing the object (Figure 2d). The LoS set generated in the region is used in the visual perception model in this method. We can formulate this LoS set S as follows (Equation (1)):

(1) $$S=\{\mathrm{LoS}\left(\emptyset_{i},\;\emptyset_{j}\right)|\;i=1,\;\ldots ,\;I,\;j=1,\;\ldots .,\;J\;and\;\emptyset_{i}=\frac{\theta_{azimuth}}{I}\left(rad\right),\;\emptyset_{j}=\frac{\theta_{elevation}}{J}\left(rad\right)\}.\;$$

If there is an obstacle in the visual area, the LoS is blocked by the object and the length of the line is reduced. Accordingly, the degree of perception varies depending on the obstacle, which means that as the volume of the visual field decreases, more obstacles exist in the visual field. Therefore, we replace the LoS with a volume. Because one line corresponds to one segment of a hemisphere, the volume of that segment area is proportional to the sum of the cube of the LoS distance in Equation (2):

(2) $$Volume\;of\;LoS_{i}=\alpha \times {\left(length\;of\;LoS_{i}\right)}^{3},$$

where α is a constant used to calculate one segment of a hemisphere. Thus, if the whole volume is divided into N pieces, this volume is a sum of one segment of a hemisphere expressed in Equation (3) according to the volume of the visible space in a stationary state:

(3) $$\begin{array}{ll} Total\;Volume & =\frac{4}{6}\times \pi \times {\left(Sight\;distance\right)}^{3}\\ & =\;N\times \alpha \times {\left(Sight\;distance\right)}^{3}\\ & =\overset{N}{\underset{i=1}{\sum }}Volume\;of\;LoS_{i}. \end{array}$$

In this paper, the degree of perception was derived according to assumption 4, i.e., that the maximum value of information is constant. Therefore, the visual perceptual degree of a driver (VPD) can be formulated only based on the scale of the LoS as follows:

(4) $$\mathrm{VPD}\;=\frac{\sum \left(Scale\;of\;LoS_{i}\right){\;}^{3}}{N},\;Scale\;of\;LoS_{i}=\frac{LoS_{i}}{LoS_{max}\;}\;.$$

However, there is a limit to constantly measuring visibility according to the road situation from the driver’s point of view with mobile sensor data. Notably, there are other objects on the road, e.g., cars, people, and other moving objects. If the driver’s UFOV is measured according to one model, the result may be inconsistent depending on the unexpected objects in the road. Therefore, to evaluate the visibility of the periphery of the road from the driver’s UFOV, it is necessary to remove the erroneous observer poses. For this reason, we established three models according to the driver’s visual perception characteristics.

### 3.2. Development of Three Types of Visual Perception Depending on Speed

**Figure 3:**
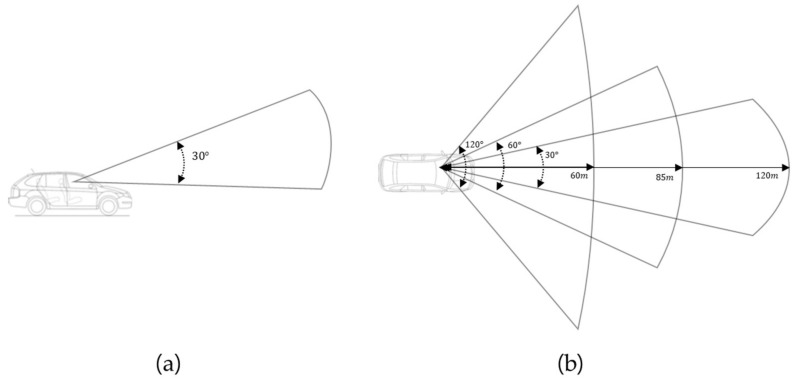
Three types of visual perception depending on speed. (**a**) Vertical visual angle of all types of perception fixed at 30°. (**b**) Three types of visual perception models according to vehicle speed.

This study adopts three types of visual perception. Figure 3 illustrates the geometry variables, and the vertical visual angle of all types of perception is fixed at 30°. For the first type, the speed is 100 km/h, the visual angle is 30°, and the sight distance is 120 m. The second type has a speed of 60 km/h, a visual angle of 60°, and a sight distance of 85 m. The third type has a visual angle of 120 degrees and a sight distance of 60 m when the speed is 40 km/h.

### 3.3. Classification of Three Cases of Road Conditions

**Figure 4:**
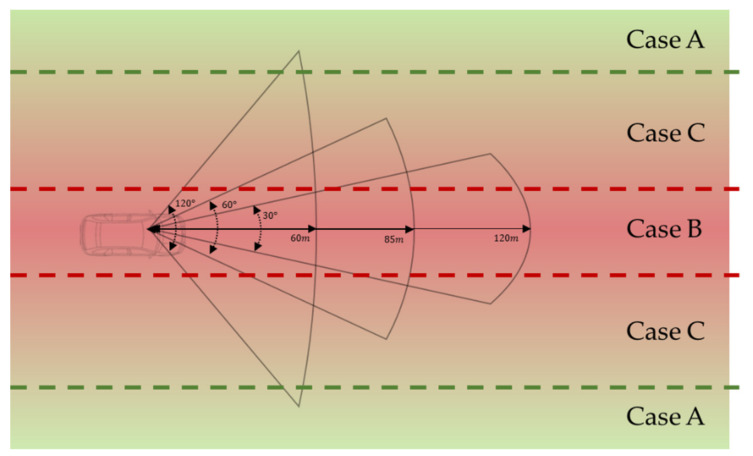
Classification of three cases of road conditions according to the visual perceptual degree (VPD) of a driver.

This study assumes that a road is basically open in the driving direction and that a driver’s view is only obstructed by road features. When the viewing angle becomes narrower, the VPDs at the 30-degree angle, the 60-degree angle, and the 120-degree angle increase. However, since the data are freely scanned using a mobile sensor, unexpected objects in the road can inevitably be encompassed in the data. Unexpected objects in the road affect the detection of road conditions when the three types of visual perception are used to identify changes in visibility depending on speed. Accordingly, to identify the safety of road conditions for drivers depending on speed, we divided road conditions into three cases by setting appropriate thresholds of the VPD, as shown in Figure 4. A grade of one to four was assigned, where one is the safest and four is the least safe.

#### 3.3.1. Case A

Case A includes a road wider than the 120-degree angle. In the case 1 road conditions, the VPDs of all three types of visual perception based on speed are greater than 0.6. This means that the road width and the road surrounding components rarely affect the degree of visual perception of drivers. Thus, the roads in case A provide a wide visibility area and are usually safe, regardless of speed in urban areas. Since the collected data include unnecessary and moving objects in or near the road, these objects occasionally affect VPDs. To avoid this unexpected influence, the roads in case A are classified as the safest grade, namely, grade 1.

#### 3.3.2. Case B

Case B includes a narrow road for which the VPDs of all three visual perception models depending on speed are less than 0.4. In this case, in contrast to case A, the road width and the surrounding components heavily affect the driver’s degree of visual perception. The roads in case B are narrow, or many objects are detected within the driver’s view. Thus, the roads in case B are the least safe since drivers must identify many and various objects while driving in urban areas. In this case, regardless of the road shapes and features, the roads in case B are classified as the least safe grade, namely, grade 4.

#### 3.3.3. Case C

For the case C road conditions, the VPD increases as the viewing angle becomes narrower. In this case, the shape of a road affects the driver’s VPD rather than other objects. The observation points on the road in case C can be classified into appropriate grades according to the corresponding VPDs. Accordingly, it can be determined whether streets are safe. In this case, the criteria for dividing the safety grade depends on the VPD at the 120-degree angle, which is where road shapes and features mainly affect the VPD at the largest viewing angle. Accordingly, grade 4 is assigned to the observation points at which the VPD at the 120-degree angle is less than 0.4. Similarly, grade 3 is assigned to the observation points at which the VPD at the 120-degree angle is between 0.4 and 0.5; grade 2 is assigned for points at which the VPD at the 120-degree angle is between 0.5 and 0.6; and grade 1 is assigned for points at which the VPD at the 120-degree angle is larger than 0.6.

### 3.4. Voxelization for the LoS Method in the Point Cloud

The VPD of a driver captures the amount of visible area. According to Equations (3) and (4), it is necessary to implement an efficient method for analyzing the collected point cloud data. Because point cloud data consist of tens of millions of points, repeatedly checking all point clouds at every observation position would require considerable computing resources and take a long time. To overcome these issues, we seek to identify a targeting area at an observation position. Because observation positions are stored in the collected point cloud data in a chronological order, the viewing direction at a selected position is the direction from that position to an adjacent position in a sequence. The sight distance depending on the three types of visual perception is equal to the radius of the visible area at an observation point.

**Figure 5:**
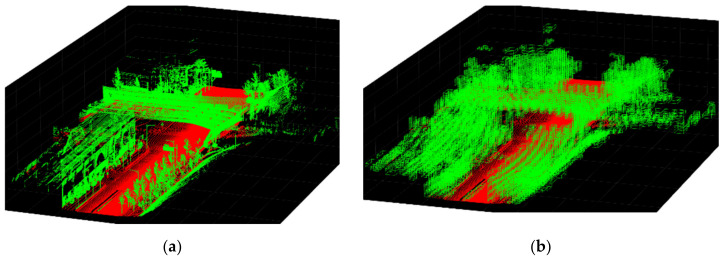
Visualization of voxelization using sample point cloud data. (**a**) Example analysis area required for each observer pose; (**b**) voxelization of point cloud data.

The collected point cloud data consist of points that do not include a volume. To capture volume information in space using a raycasting algorithm, we voxelized the point cloud data using the octree method, which has been widely adopted for analyzing large point clouds [31]. According to the octree method, point clouds are recursively divided into small voxels. The criteria to voxelize point clouds in this study are to divide a voxel if the number of points is more than 10 and to stop the division when the size of a voxel is smaller than 0.5 m. Because the voxel resolution affects the processing time in this study, we determined 10 points and 0.5 m by investigating the point resolution in the collected data such that the mobile sensor resolution effectively captured spatial forms. Figure 5 shows a visualization of voxelization using sample point cloud data.

**Figure 6:**
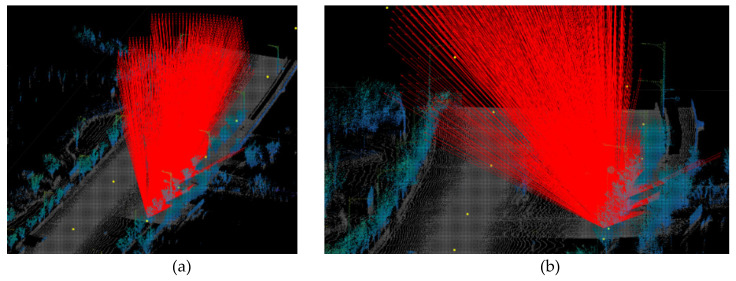
Visualization of the raycasting method with point cloud data. (**a**) Example visualization (**b**) Detailed visualization on the street with trees.

The visibility analysis can be performed within the voxelized point cloud, which makes it possible to raycast objects such as trees that are hard to virtually describe. Figure 6 illustrates the visualization of the raycasting method with the point cloud data and some trees that affect visibility in an urban space.

## 4. Experiments in an Urban Space

### 4.1. Test Site

**Figure 7:**
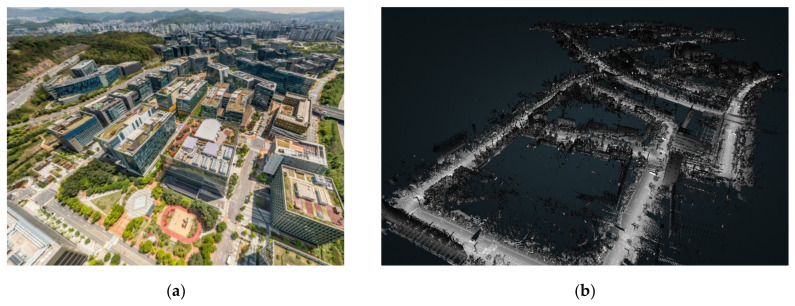
Aerial view of the test site in Pangyo, South Korea. (**a**) Aerial view (Naver Maps [33]), (**b**) Collected point cloud data [32].

**Figure 8:**
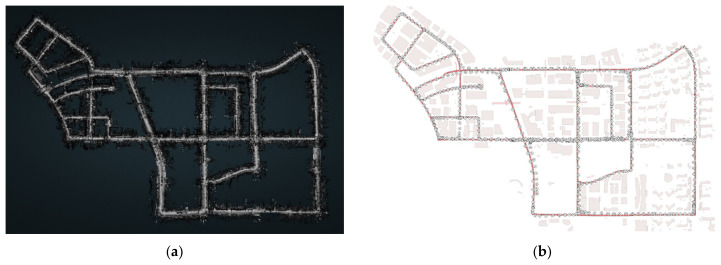
Top view of the test site in Pangyo, South Korea. (**a**) Collected point cloud data [32], (**b**) Distribution of observation positions.

In this paper, the proposed visual perception model was applied to mobile laser-scanned data from Pangyo [32] to evaluate traffic safety in the city. Figure 7a shows an aerial view of the Pangyo area and Figure 7b shows the collected point cloud of the same area. The dataset consists of 77,416,102 points in the urban space and 200,510 driver observation locations, i.e., pose locations, which are points that have been captured by a mobile scanner in the urban space. The driver observation points reflect instantaneous locations. However, due to the required computing time and resource limitations, this study has chosen 668 interval values for the driver observation locations to appropriately cover the chosen site in Pangyo. Figure 8 shows a map of the point cloud from the top view and the observation positions extracted at a certain interval with the driver’s path.

### 4.2. Model Implementation

**Figure 9:**
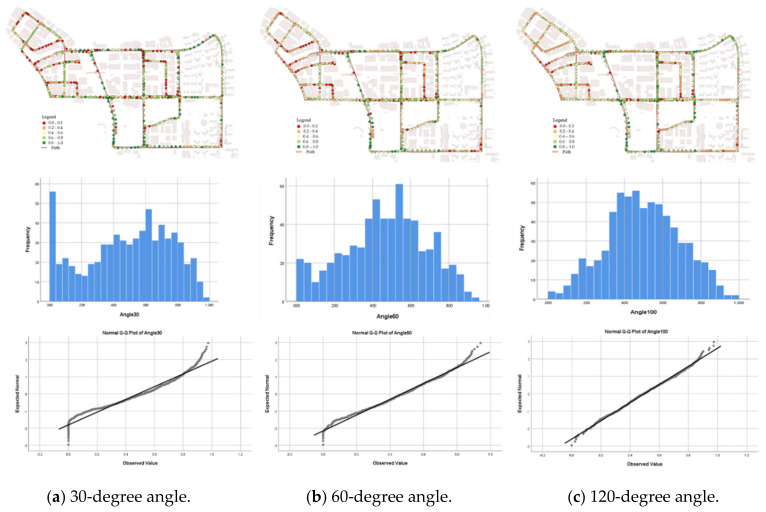
VPD results at 30-, 60-, and 120-degree angles.

The VPDs for the three types of visual perception were calculated at 668 observation positions using the point cloud data collected in Pangyo. The top images in Figure 9 show the VPDs for 30-, 60-, and 120-degree angles at each observation position. As the viewing angle decreases (i.e., the speed increases), the VPDs include more values that rarely follow a normal distribution. At small viewing angles, abnormal values become more frequently observed in urban areas. Normality tests were carried out to determine whether the VPDs for the 30-, 60-, and 120-degree angles at each observation position would be feasible to assess road conditions. According to the three normality tests, i.e., the Kolmogorovؘ–Smirnov test [34,35], Anderson–Darling test [35,36], and Shapiro–Wilk test [35,37], VPDs at a 120-degree angle follow the normal distribution in Table 1 at a confidence level of 95%, except in the case of the Shapiro–Wilk test. However, since the Shapiro–Wilk test is sensitive to the number of samples, it is suggested to use the normality test instead of the Shapiro–Wilk test when the number of samples does not exceed 50 [37,38]. According to the histogram and the frequency distribution chart, unexpected VPDs are more frequently measured at the 30-degree angle than at the other angles. Because VPDs at the 120-degree angle are normally distributed, VPDs at the 120-degree angle can be effectively used to determine the road safety at each observation position.

**Figure 10:**
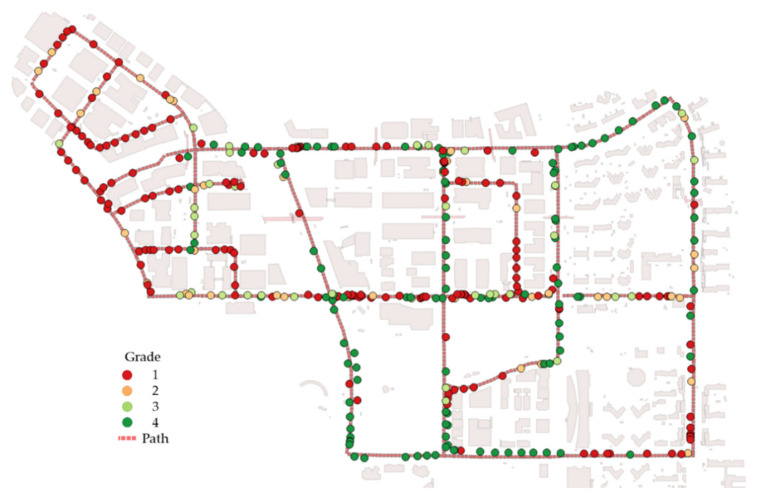
Distribution of grades 1 to 4 at the observation positions.

Accordingly, VPDs were calculated in Pangyo. Figure 10 shows the distribution of grades at observation points. Each observation position was evaluated based on grades 1 to 4. The red dots are grade 1; the yellow dots are grade 2; the light green dots are grade 3; and the dark green dots are grade 4.

### 4.3. Analysis

**Figure 11:**
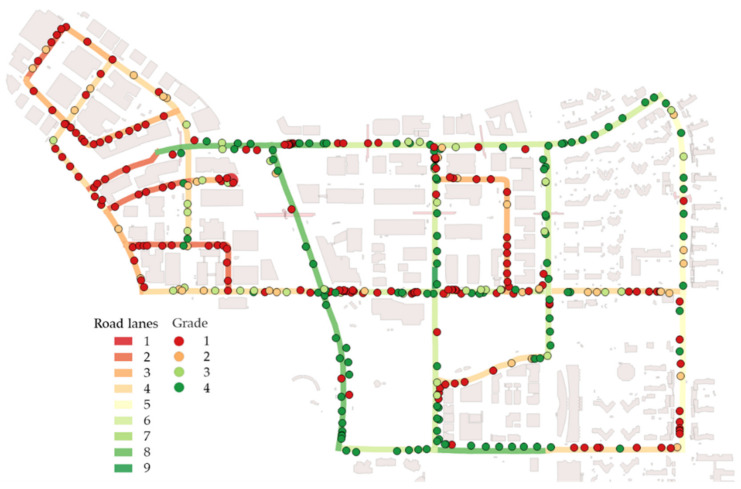
Comparison of grades at observation positions and the number of road lanes.

According to the distribution of grades 1 to 4, road safety usually reflects the road width, i.e., the number of lanes. Figure 11 shows a comparison of grades at observation positions and the number of lanes.

**Figure 12:**
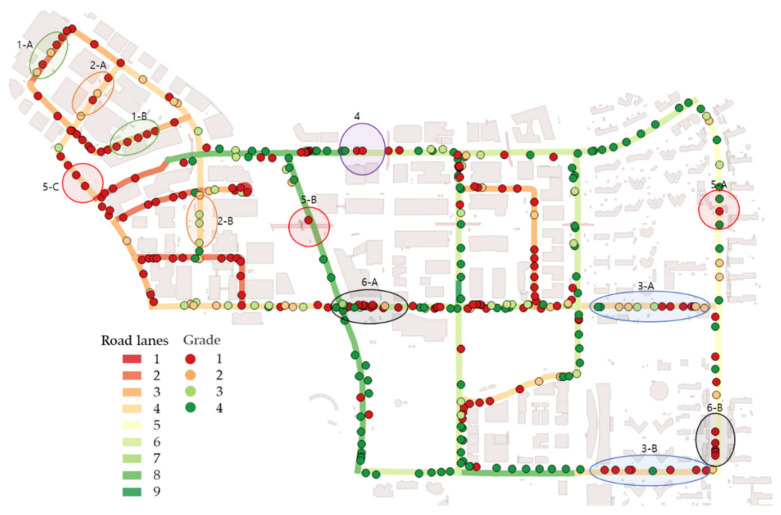
Locations of six zones identified in Pangyo.

By comparing grades, the number of road lanes, and building polygon data obtained from a Korean government website (the Korea National Spatial Data Infrastructure Portal www.nsdi.go.kr), we identified six groups in this area. Although the grades at the majority of the observation positions correspond to the number of road lanes, the grades at some positions are different from those at nearby positions. In the following section, we seek to identify why such differences occur (see Figure 12).

## 5. Discussion

We investigated why the six groups occur in this area. By obtaining and investigating road-view panoramic images from Naver maps [32], we identified road shapes and features that affect the VPD of a driver at observation positions in Pangyo. Accordingly, we identified features such as the tree density, building layout, open space, construction of new buildings, overpasses, and moving objects on roads in Figure 12.

### 5.1. Tree Density

**Figure 13:**
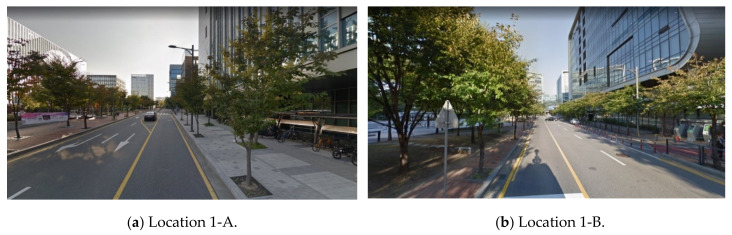
VPD difference according to the tree density (Naver Maps [33]).

The tree density affects driver visibility. Although the road widths and positions of buildings are similar at locations 1-A and 1-B, the VPDs are different. According to the street view in Figure 13, dense trees exist at location 1-B. Because dense trees obscure drivers’ views, the risk of road accidents increases.

### 5.2. Building Layout

**Figure 14:**
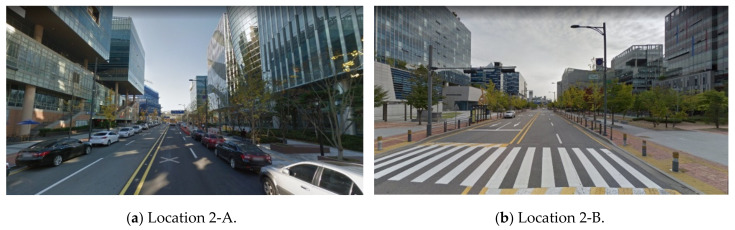
VPD difference according to the building layout (Naver Maps [33]).

The layout and density of buildings affects drivers’ visibility on the road. Although the locations of 2-A and 2-B have similar road widths and building positions, the VPDs are different. According to the street view in Figure 14, buildings at location 2-A are closer to the road, and the buildings are larger than those at location 2-B. Although some buildings along both roads have similar building footprints, the spaces between the roads and buildings are different.

### 5.3. Open Space

**Figure 15:**
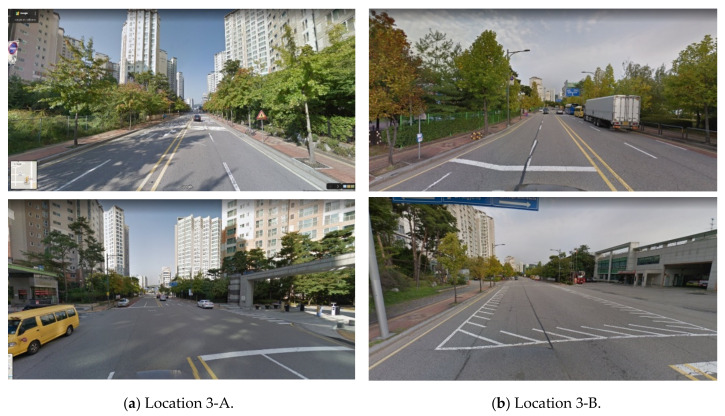
Sudden VPD changes as open space appears (Naver Maps [33]).

Open space affects drivers’ visibility in urban areas. Even if the road width and layout are constant, open spaces such as entrances and parks may exist. In this case, the driver’s view suddenly expands, resulting in a high VPD. According to the comparison of the street views at locations 3-A and 3-B in Figure 15, although the vegetation and buildings are arranged in a row, the visible area suddenly changes at the entrance to the apartment complex at location 3-A and at the fire station at location 3-B. Accordingly, if the VPD suddenly increases, it is necessary to focus on road safety.

### 5.4. New Buildings

**Figure 16:**
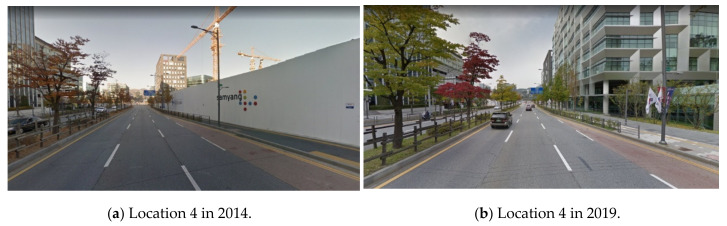
New building found at location 4 (Naver Maps [33]).

Since this study analyzes point cloud data collected with a mobile scanner, VPDs reflect up-to-date road features. At location 4, no buildings exist, but according to the street view recently obtained at location 4, a new building is being constructed in Figure 16. Since the building polygon data were created in 2015, the space at location 4 is empty on the map, and the VPD is expected to be high. However, VPDs and driver visibility are influenced by new buildings. Accordingly, when we analyze up-to-date point cloud data collected by a mobile scanner, it is possible to quickly and realistically evaluate road safety.

### 5.5. Overpasses

**Figure 17:**
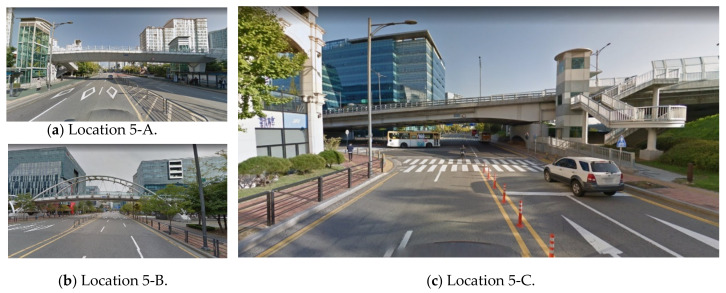
Overpasses at which the VPD suddenly changes (Naver Maps [33]).

Overpasses affect driver visibility in urban areas. In particular, the VPDs for three-dimensional urban features can be calculated because the collected point cloud data consist of three-dimensional elements. In particular, overpasses are significant road features that affect drivers’ views. Similar to trees, overpasses are rarely identified in two-dimensional (2D) geolocational data and constructed virtual environments. Since the point cloud data are collected by a mobile scanner, it is relatively easy to identify overpasses. According to Figure 17, the overpass affects VPDs at the location show, with lower VPDs than at nearby observation positions.

### 5.6. Moving Objects

**Figure 18:**
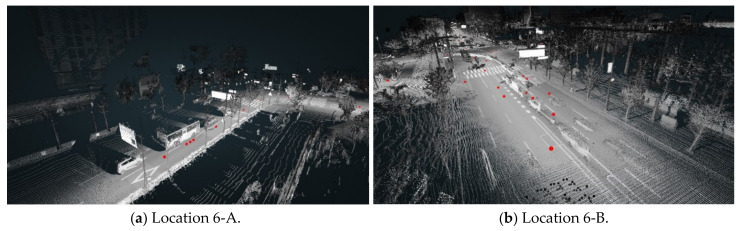
Unexpected VPD results due to moving objects.

Although we considered moving objects in the VPD calculation, some VPDs are highly influenced by moving objects. Because a moving object on a road is scanned by a mobile scanner, some exceptions may occur. Although the road consists of eight lanes at location 6-A, the VPD is low due to moving objects, i.e., the vehicles in Figure 18a. A moving car at the intersection was captured by a mobile scanner at location 6-B, and this car obscures the driver’s view at the observation position in Figure 18b. At locations 6-A and 6-B, the VPDs are low regardless of the road shape and features. To avoid these unexpected findings, it is necessary to scan targeted areas when no or few vehicles are on the road.

## 6. Conclusions

In this study, we have proposed a method for evaluating road safety by analyzing point cloud data collected by a mobile scanner. By developing three types of visual perception depending on vehicle speeds, we have identified locational candidates that require further investigation. By analyzing point cloud data with the proposed method, we have verified that trees and obscuring objects along a road affect drivers’ visibility. Therefore, the proposed method is helpful for evaluating realistic and various road safety conditions. However, it is necessary to develop a method to quickly and consistently classify road safety grades. Additionally, in order to prevent errors caused by unnecessary road elements, it is suggested to adopt the proposed method to evaluate road safety when moving objects become least prevalent in urban areas, such as at midnight or dawn.

This study has developed a method to analyze urban environments and the visual perception of real, sensor-collected physical forms in urban areas. In particular, this study has demonstrated changes in perceiving elements of roads and urban physical contexts. In order to adopt the proposed method in a large urban area, further studies are suggested. Since this study analyzes a district in Pangyo, Korea, it would be necessary to examine whether roads in other cities can be assessed with the proposed method. Further statistical analysis and conjecture on the extrapolation of this evaluation to the larger road networks of a city or a country would provide guidance on how to extract results for more theoretical analyses and practical applications. In addition, testing actual perceived elements on roads with human eyes would be helpful to generalize the proposed method in order to assess road safety. A further study would allow for the experiment in this study to be replicated in various urban environments of other cities.

## Figures and Tables

**Table 1:** Test of normality.

Test of Normality						
Viewing Angle	Kolmogorov–Smirnov		Shapiro–Wilk		Anderson–Darling	
	Statistic	*p*-Value	Statistic	*p*-Value	Statistic	*p*-Value
30 degrees	0.070	0.000	0.954	0.000	7.619	0.000
60 degrees	0.037	0.034	0.984	0.000	1.849	0.000
120 degrees	0.031	0.195	0.995	0.027	0.528	0.170
